# Verification of a Dream

**Published:** 1886-09

**Authors:** 


### *VERIFICATION OF A DREAM.*

The New York *Sun* of a recent date gives a circumstantial account of the discovery of a chest containing $27,000 in gold and silver coin near the old fort in the little town of Franklin, Pa. For many years it had been a current belief that at the time of the hasty evacuation by the French of the fort, a large amount of treasure was secretly buried by the retreating forces somewhere in its near vicinity.

It seems that Columbus Brown, ot Franklin, had for years held to the belief that, through some means, he was to become possessed of this treasure.

About two years ago two Frenchmen, supposed to be relatives to the old commandant of the fort, arrived at Franklin with maps and commenced a systematic search, but it proved fruitless. Brown became excited at this, and since that time has continued the search. One night recently, while sleeping, he had a revelation. He dreamed that he was handling and counting a chest of gold, and that he had found it buried in the earth at the foot of a tree, in an open field. He was informed in some manner, he cannot tell how, but by a man with a foreign accent, dressed in a military uniform, with a sword and sash, that if he would measure a certain distance from the centre of a rock in the run, due north, and then measure thirty-three feet due west from that point, he would find the treasure he had so often seen in his dream. He arose, and with spade and pick went to the owner of the field in which the tree stood and gained permission to dig. He had not been at work more than two hours when he came upon an iron chest, which he opened, and the sight that met the gaze of himself and son was enough to turn the head of almost any man. The box was nearly two-thirds filled with gold and silver coin, tarnished and covered with sand and mould.

The coins are mostly French, but a number of English, German and Spanish coins are among the lot. They bear the dates 1729, 1744, 1751, and various other dates, the latest of which is 1754, which is the same year that Fort Macnault was completed. On a brass ruler found in the chest, the name "Foncaire" is plainly stamped. It is a well-known fact that this was the name of the officer in command of the French troops. The fort was evacuated in July, 1759, and very hastily. The location of this field is about seventy-five rods south of the fort, and was no doubt selected for the burial of the treasure with a view to mislead the Indians and recovering it at a subsequent date. Mr. Brown took the chest to his home, and many of the coins have since been on exhibition in the banks.

